# Effects of Bee Venom Injections at Acupoints on Neurologic Dysfunction Induced by Thoracolumbar Intervertebral Disc Disorders in Canines: A Randomized, Controlled Prospective Study

**DOI:** 10.1155/2015/363801

**Published:** 2015-11-29

**Authors:** Li-Chuan Tsai, Yi-Wen Lin, Ching-Liang Hsieh

**Affiliations:** ^1^Graduate Institute of Acupuncture Science, College of Chinese Medicine, China Medical University, Taichung 40402, Taiwan; ^2^Research Center for Chinese Medicine & Acupuncture, China Medical University, Taichung 40402, Taiwan; ^3^Graduate Institute of Integrative Medicine, College of Chinese Medicine, China Medical University, Taichung 40402, Taiwan; ^4^Department of Chinese Medicine, China Medical University Hospital, Taichung 40447, Taiwan

## Abstract

Intervertebral disk disease (IVDD) is a major spine disorder in canines that causes neurological dysfunction, particularly in the thoracolumbar area. Analgesic and anti-inflammatory drugs are typically used to reduce nociceptive signals to decrease canine suffering. Bee venom (BV) has been reported to exert anti-inflammatory and analgesic effects. Injection of BV at acupoints has been widely used to treat clinical disorders including inflammation, pain, and arthritis. The current study was intended to determine whether BV injections at acupoints can enhance treatment of canine neurological dysfunction caused by IVDD. A single-blind controlled trial involving 40 adult canines with neurological dysfunction induced by IVDD subdivided into 2 groups was designed, and 36 canines finished the study. The myelopathy scoring system (MSS) grade and functional numeric scale (FNS) scores improved further after BV treatment than after control treatment. BV injection exerted a particularly strong effect on canines with moderate to severe IVDD and dramatically reduced clinical rehabilitation time. The results indicate that BV injections at acupoints are more effective at protecting canines from IVDD-induced neurological dysfunction and pain than is treatment alone.

## 1. Introduction

Intervertebral disc disease- (IVDD-) induced neurological dysfunction is one of the spinal diseases most frequently diagnosed in clinics with a prevalence of approximately 2% [1, 2]. IVDD in dogs is often observed in the thoracolumbar section with degenerated and abnormal discs, causing neurological deficit and neuropathic pain. In clinical settings, paresis or paralysis and increased urinary retention invariably accompany IVDD-induced hyperalgesia [3]. Surgery is a well-established method for treating IVDD, particularly in acute and severe cases. Oral administration of anti-inflammatory medications, analgesics, and steroids is another method for treating IVDD.

Bee venom (BV) is used for long-term treatment of inflammation, pain, and arthritis. Several compounds are purified from BV, such as melittin, adolapin, apamin, phospholipase, and amine. These compounds can potentially cure inflammation, reduce pain, and treat arthritis [4]. BV injected into Zusanli (ST36) acupoints can decrease chronic constrictive injury of sciatic nerve-induced neuropathic pain by modulating *α*2-adrenoceptors [5]. Scientists have also reported that BV injections inhibit immune response and anti-inflammation in type 2 collagen-induced arthritis [6]. Lee et al. suggested that BV can alleviate pain symptoms caused by rheumatoid arthritis and osteoarthritis [7].

Decompression surgery is typically used to treat IVDD, using hemilaminectomy or dorsal laminectomy [8]. Kinzel et al. reported that partial percutaneous discectomy can reliably reduce thoracolumbar disc protrusion with a recovery rate of 88.8% [9]. Physical therapy, such as swimming, massaging, and towel walking, is often used to attenuate IVDD [10]. Numerous studies have indicated that acupuncture is effective for treating low back, inflammatory, neuropathic, and postoperative pain [8, 11–16]. The advantage of using acupuncture as an alternative or complementary therapy is convenience and low cost. Acupuncture can trigger the release of endogenous enkephalin, endorphins, dynorphins, and adenosine. Currently, the effects and mechanisms of BV injections at acupoints remain unknown.

Recently, acupuncture has been widely used for treating IVDD in canines [8, 10, 16], but researchers remain unsure whether BV injections can produce similar effects. In this study, we hypothesized that BV injected at acupoints can relieve IVDD-induced pain signals in dogs. Our study population included 36 adult canines with neurological dysfunction caused by IVDD. Our results indicated that neurological scores improved more from weeks 2 to 6 in the BV injection group than in the control group. Our results provide novel evidence that BV injections at acupoints are crucial for treating IVDD-related neurological dysfunction and pain.

## 2. Material and Methods

### 2.1. Animals

We used X-ray examination to identify IVDD in the thoracolumbar section of all canines in the study population. We presumptively diagnosed thoracolumbar disc protrusion or extrusion based on clinical signs, history, and radiographic physical examination. We also performed myelography or computed tomography (CT) examinations when necessary for diagnosis. Furthermore, specialized veterinarians evaluated all canines for IVDD-induced neurological dysfunction. All procedures were approved by the Institute of Animal Care and Use Committee of China Medical University (permit no. 101-116-N; 2010, 08) and were in accordance with the* Guide for the Use of Laboratory Animals* published by the National Research Council.

The study was conducted in 3 private animal hospitals in Taichung, Taiwan, between August 2010 and August 2012.

### 2.2. Inclusion and Exclusion Criteria

The study included canines of both sexes aged 1 to 8 years. Various breeds of canine were included. Canines with the following symptoms were excluded from this study: (1) severe diseases such as heart failure, liver failure, kidney failure, encephalitis, bacterial infection, and viral infection, for which steroid therapy is unsuitable; (2) neurological dysfunction caused by trauma, degenerative spinal diseases, discospondylitis, and tumors.

### 2.3. Grouping

We observed 40 canines, randomly assigned to either the control group or the experimental group based on the order in which medical advice was sought. Each group contained 20 canines. Canines in the control group received standard treatment, oral prednisone (1 mg/kg/day) together with the nonsteroidal anti-inflammatory drug (NSAID) carprofen (2.2 mg/kg/day) for 7 days. Ranitidine (2 mg/kg/day) for 5 or 7 days also was administered to prevent gastrointestinal disturbance. Antibiotics were administered after performing a urinary antibiotic sensitivity test. Canines in the experimental group were injected with BV in addition to standard treatment. We injected BV (Guju Pharmacological Co. and APIMEDS Co., Korea) at bilateral LI 04, SI 03, KI 03, ST 36, BL 23, BL 40, GB 30, GB 34, and LR 03, unilateral GV 01, Baihui, and Ashi points [8, 12, 16]. The BV injection solution contained the following: Apimellena (main ingredient: Apitoxin) at 5 mg/vial diluted using 6.2 mL of saline, 0.4 mL of Apitoxin (400 *μ*g), and 0.4 mL of Lidocaine (8 mg). We sterilized each acupoint by using alcohol and then injected 0.1 mL (20 *μ*g) of BV solution into the acupoint, twice a week for 6 weeks.

### 2.4. Assessments

A well-trained veterinarian who was blind to the grouping assessed each canine for neurological deficit. The assessment was performed before treatment and at 1 week (7 ± 3 days), 2 weeks (14 ± 3 days), 3 weeks (21 ± 3 days), 4 weeks (28 ± 3 days), 5 weeks (35 ± 3 days), and 6 weeks (42 ± 3 days) after treatment.

Severity of neurological deficit was evaluated according to the myelopathy scoring system (MSS) grade [8, 12, 17–19]. The scoring system is as follows: Grade 1: focal pain only; Grade 2: able to bear weight, deficits of proprioception, and ambulatory paraparesis; Grade 3: unable to bear weight, severe incoordination, intact spinal reflexes or hyperreflexia, and deep pain perception; Grade 4: nonambulatory paraparesis, deficits of proprioception, and deep pain perception; Grade 5: any of the aforementioned clinical signs in addition to paraplegia, no deep pain perception, and bladder dysfunction. Functional numeric scale (FNS) scores, as described by Hayashi et al. [10], were also used. The highest possible score was 23, including 4 points for ability to stand, 4 points for movement of pelvic limbs, 4 points for deep pain perception, 4 points for urinary control, 4 points for ability of walk, and 3 points for movement of the tail.

### 2.5. Statistical Analysis

All statistical data are presented as the mean ± standard error. Statistical significance between each group was tested using a chi-square test, independent *t*-test, and paired *t*-test (*P* < 0.05 was considered statistically significant).

## 3. Results

**Figure 1:**
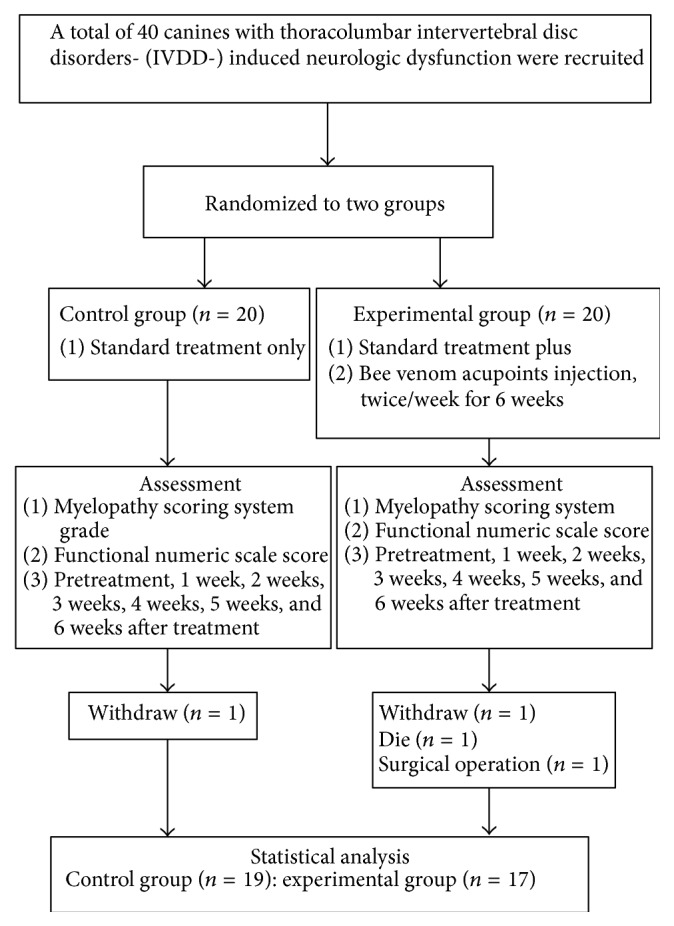
Flowchart of canines involved in this study.

Forty canines met criteria into the trial. One canine in the control group was withdrawn from the study. In the experimental group, one canine was withdrawn, one canine died, and one canine underwent surgery (Figure 1). Therefore, a total of 36 (19 in the control group and 17 in the experimental group) canines finished the 6-week study. We first compared the basic information of the 2 groups. The data showed no significant difference in sex, age, weight, or MSS grade (all *P* > 0.05; Table 1).

The results indicated that the FNS score of the control group was 10.84 ± 9.14 before treatment. After treatment, the FNS score increased to 13.58 ± 9.5 in all canines from Grades 1 to 5 (Table 2, *P* = 0.002, *n* = 19). The MSS grade improved after treatment (3.42 ± 1.5 before treatment and 2.74 ± 1.49 after treatment, *P* = 0.001, *n* = 19; Table 2).

We further subdivided the canines into mild (Grades 1-2), moderate (Grades 3-4), and severe (Grade 5) subgroups to confirm that the treatment was effective for all grades. Based on the FNS scores, we determined that the control treatment exerted a curative effect in the mild group (from 21 ± 1.73 to 22.57 ± 0.78, *P* = 0.017, *n* = 7; Table 2). The MSS grade indicated similar results (from 1.71 ± 0.48 to 0.71 ± 0.95, *P* = 0.017, *n* = 7; Table 2). Furthermore, our results revealed that the control treatment exerted no effect on the canines with moderate IVDD (from 10.64 ± 4.03 to 16.2 ± 6.38, *P* = 0.074, *n* = 5; Table 2). The MSS grade also did not differ significantly before and after control treatment (from 3.6 ± 0.54 to 2.8 ± 1.09, *P* = 0.099, *n* = 5; Table 2). We next checked whether the control treatment reduced FNS and MSS scores in the canines with severe IVDD. The FNS score demonstrated that the control treatment did not effectively treat clinical symptoms (from 0.86 ± 0.37 to 2.71 ± 2.28, *P* = 0.073, *n* = 7; Table 2), and the MSS grade indicated similar results (from 5 to 4.71 ± 0.48, *P* = 0.172, *n* = 7; Table 2).

We next used BV injections at acupoints to determine whether BV can enhance the control treatment for canines with IVDD. The FNS score was 11.71 ± 8.16 before treatment and increased to 19.41 ± 5.87 after treatment in all canines (*P* = 0.001, *n* = 17; Table 2). The MSS grade was 3.41 ± 1.32 before treatment and decreased to 1.24 ± 0.6 after BV injection (*P* = 0.001, *n* = 17; Table 2). We also analyzed and divided all canines into mild (Grades 1-2), moderate (Grades 3-4), and severe (Grade 5) of 3 groups compared to control treatment. Our results identified that BV injection caused significant reduction of both FNS score and MSS grade. In the mild group, our results indicated that BV injection at acupoints significantly reduced FNS score (from 20.6 ± 1.52 to 23, *P* = 0.024, *n* = 5; Table 2) and MSS grade (from 1.80 ± 0.45 to 0.00, *P* = 0.001, *n* = 5; Table 2). Series results were also obtained in other groups. We also observed that BV injections substantially increased FNS score (from 12.57 ± 4.89 to 22.71 ± 0.48, *P* = 0.001, *n* = 7; Table 2) and MSS grade (from 3.43 ± 0.53 to 0.98 ± 0.57, *P* = 0.001, *n* = 7; Table 2) in the moderate group. The results for the severe group also indicated that BV injections reliably increased the FNS score (from 1.6 ± 0.89 to 11.4 ± 4.61, *P* = 0.014, *n* = 5; Table 2). The MSS grade also improved after BV treatment (from 5 to 3.4 ± 0.55, *P* = 0.002, *n* = 5; Table 2).

**Figure 2:**
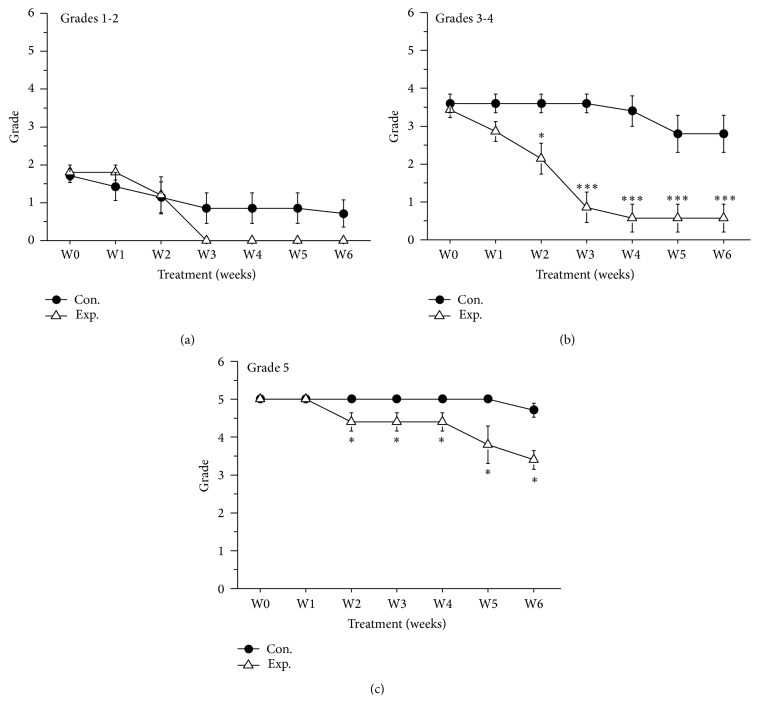
MSS grades of the control and experimental groups. (a) MSS grades of control and experimental canines with mild IVDD (Grades 1 and 2). (b) MSS grades of control and experimental canines with moderate IVDD (Grades 3 and 4). (c) MSS grades of control and experimental canines with severe IVDD (Grade 5). *∗* indicates *P* < 0.05, compared with the control group. *∗∗∗* indicates *P* < 0.001, compared with the control group.

**Figure 3:**
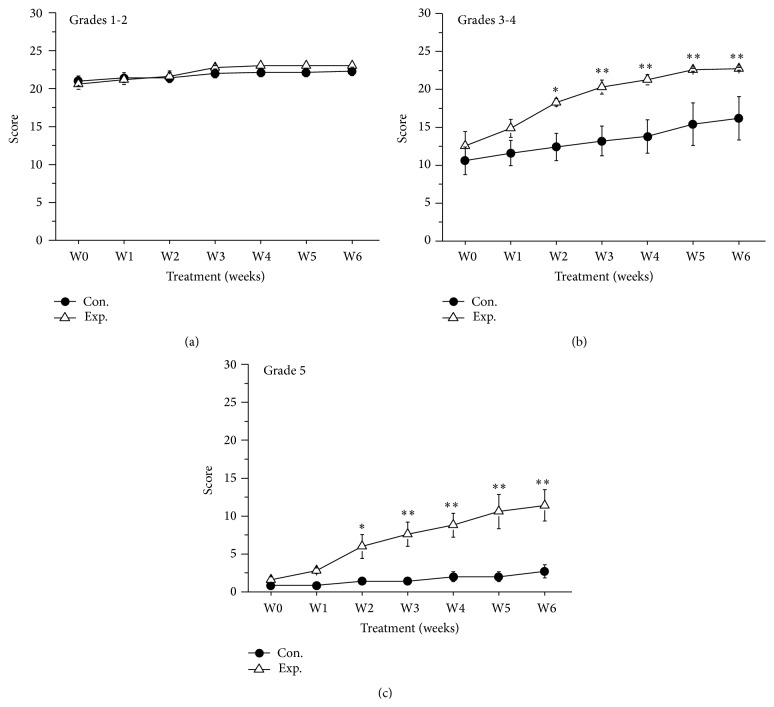
FNS scores of the control and experimental groups. (a) FNS scores of control and experimental canines with mild IVDD (Grades 1 and 2). (b) FNS scores of control and experimental canines with moderate IVDD (Grades 3 and 4). (c) FNS scores of control and experimental canines with severe IVDD (Grade 5). *∗* indicates *P* < 0.05, compared with the control group. *∗∗* indicates *P* < 0.01, compared with the control group.

We further compared the therapeutic effects on the control group and experimental group over time. The MSS grades of canines with mild IVDD in both the control group and experimental group indicated that treatment reversed the effects of IVDD and that the canines exhibited maximal response to treatment after 2 weeks (Figure 2(a), *P* > 0.05). Compared with control treatment alone, BV injections significantly reduced the MSS grade of canines with moderate IVDD at 2 weeks after administration (Figure 2(b), *P* < 0.05). These results indicated that BV injections are a novel, more effective therapy for IVDD in canines (Figure 2(c), *P* < 0.05). FNS scores of the 3 IVDD severity subgroups exhibited similar results. In canines with mild IVDD, both control treatment and BV treatment caused weekly increases in FNS score (Figure 3(a), *P* > 0.05). In canines suffering from moderate IVDD, control treatment improved the FNS score (Figure 3(b), *P* > 0.05). For canines with severe IVDD, BV treatment dramatically facilitated the recovery of IVDD at 2 weeks after treatment (Figure 3(c), *P* < 0.05). Overall, BV treatment is effective for treating severe IVDD in canines. Based on the aforementioned results, BV injections at acupoints are extremely effective for treating IVDD in canines.

We next determined whether BV injections can reduce repair time (RT) in veterinary clinics. We calculated the RT values of canines of all MSS grades. In the control group, the RT was 4.89 ± 1.85 weeks; in the BV group, the RT was 4.24 ± 1.43 weeks (Table 3, *P* > 0.05). In canines with mild IVDD, the RT value was similar in both the control treatment and BV treatment canines (3.14 ± 2.12 and 2.6 ± 0.54 for the control group and BV group, resp., *P* > 0.05; Table 3). In canines with moderate IVDD, the RT in the control group was 5.2 ± 0.84 weeks; in the BV treatment group, RT decreased to 3.0 ± 0.58 weeks (Table 3, *P* = 0.001). BV injections also reduced the RT in canines with severe IVDD from 6 weeks in the control group to 5.4 ± 0.55 weeks in the BV group. These results indicated that BV injections at acupoints benefit canines with IVDD.

## 4. Discussion

This paper reports that both control treatment and BV treatment can reliably improve the MSS grade and FNS score of canines with IVDD. Control treatment can slightly reduce MSS and FNS values of canines with mild IVDD but is ineffective for moderate to severe IVDD. BV injections at acupoints substantially improved the MSS and FNS scores of canines with moderate to severe IVDD. Furthermore, BV injections reduced the RT of canines with moderate to severe IVDD, suggesting that they are an effective form of therapy. Differences between control treatment and BV treatment appeared at 2 weeks after BV administration. Our data indicated that BV injections at acupoints have the potential to become a novel clinical treatment for canines with IVDD.

Previous studies have suggested that treatment is considered successful if it can control pain or improve proprioception or ataxia in cases of mild IVDD. Similarly, treatment must enable the animal to walk without support [8]. Our results indicated that BV injections can improve both MSS and FNS scores in canines with moderate to severe IVDD, meaning that BV is an effective form of therapy. We also observed that the repair rate in canines with mild-to-moderate IVDD that received control treatment was 75%, which is similar to previous studies that have indicated repair rates of 54.7% [20] and 67.6% [11]. Previous studies have indicated that, in cases of severe IVDD, with nearly complete loss of nerve system function, repair time can extend to 6 months [8], and in some cases recovery might not be possible [10]. Our study demonstrated that BV injections at acupoints exert a therapeutic effect on canines with severe IVDD, which indicates the potential of BV injections for clinical IVDD treatment, particularly in veterinary settings.

Decompression surgery is often considered as a major form of therapy for cases of severe IVDD [21, 22]. The recovery rate for acute mild-to-moderate IVDD is 93% to 95% if decompression surgery is performed within 48 hours. In cases of severe IVDD, the recovery rate decreased to 43% [8]. Previous research indicated that electroacupuncture is a more effective form of therapy than decompression surgery only for canines that have not received decompression surgery within 48 hours [8]. Several articles demonstrated that acupuncture and electroacupuncture shorten the IVDD recovery period [8, 10, 16, 21]. Our results indicated that BV injections at acupoints reverse 100% of cases of mild IVDD in canines, compared with other studies that have indicated an average recovery rate of 97% to 100% [10, 16]. Furthermore, the repair rate of canines that received BV injections is close to 100%, which is similar to electroacupuncture [7] therapy and superior to acupuncture [10, 16]. BV injections can trigger the endogenous pain inhibitory system to release neurotransmitters or neuropeptides to reduce pain signaling. Several studies have demonstrated that BV injections can cause the pituitary gland and adrenal gland cortex to increase the concentration of cortisol [23–25]. BV can also activate opioid receptors and *α*2-adrenoceptors [5] and the descending serotonergic pathway [26]. Our results indicated that BV injections at acupoints can relieve IVDD symptoms and particularly aid in the recovery of moderate to severe IVDD in canines.

## 5. Conclusion

This study showed that BV injections at acupoints can successfully improve MSS and FNS scores. The RT of canines that received BV injections was shorter than that of canines that received only control treatment, particularly in cases of moderate to severe IVDD. We further concluded that BV injections are an easy, convenient, and effective form of therapy for canines with IVDD.

## Figures and Tables

**Table 1:** Basic comparison in control and experimental groups.

	Control	Experimental	*P* value
	(*n* = 17)	(*n* = 19)	
Gender			0.342
Male	13 (68.4%)	9 (52.9%)	
Female	6 (31.6%)	8 (47.1%)	
Age (y)	6.52 ± 1.68	6.59 ± 1.69	0.898
Weight (kg)	6.95 ± 4.92	7.78 ± 3.48	0.570
MSS grade			0.896
1	2 (10.5%)	1 (5.9%)	
2	5 (26.3%)	4 (23.5%)	
3	2 (10.5%)	4 (23.5%)	
4	3 (15.8%)	3 (17.6%)	
5	7 (36.8%)	5 (29.4%)	

MSS grade: grade of myelopathy scoring system.

**Table 2:** Effects of Bee venom on FNS and MSS in canines with intervertebral disc disorders-induced neurologic dysfunction were recruited.

Grade	FNS		*P* value	MSS		*P* value
	Before	After		Before	After	
Control						
1–5 (*n* = 19)	10.84 ± 9.14	13.58 ± 9.50	0.002	3.42 ± 1.50	2.74 ± 1.94	0.001
1-2 (*n* = 7)	21.00 ± 1.73	22.57 ± 0.78	0.017	1.71 ± 0.48	0.71 ± 0.45	0.017
3-4 (*n* = 5)	10.64 ± 4.03	16.20 ± 6.38	0.074	3.60 ± 0.54	2.80 ± 1.09	0.099
5 (*n* = 7)	0.86 ± 0.37	2.71 ± 2.28	0.073	5.00	4.71 ± 0.48	0.172
Experimental						
1–5 (*n* = 17)	11.7 ± 8.16	19.41 ± 5.87	0.001	3.41 ± 1.32	1.24 ± 0.60	0.001
1-2 (*n* = 5)	20.60 ± 1.52	23.00	0.024	1.80 ± 0.45	0.00	0.001
3-4 (*n* = 7)	12.57 ± 4.89	22.71 ± 0.48	0.001	3.43 ± 0.53	0.98 ± 0.57	0.001
5 (*n* = 5)	1.60 ± 0.89	11.40 ± 4.61	0.014	5.00	3.40 ± 0.55	0.002

Control: control group; experimental: experimental group; before: before treatment; after: after treatment; FNS: functional numeric scale; MSS: myelopathy scoring system; grade: grade of MSS.

**Table 3:** Comparison in repair time between control and experimental groups (weeks).

Grade	Control	Experimental	*P* value
1–5	4.89 ± 1.85	4.24 ± 1.43	0.245
1-2	3.14 ± 2.12	2.60 ± 0.54	0.59
3-4	5.20 ± 0.84	3.00 ± 0.58	0.001
5	6	5.40 ± 0.55	0.014

Mean ± standard deviation. Grade: grade of myelopathy scoring system; control: control group; experimental: experimental group.
